# Impact of ICU-acquired weakness on post-ICU physical functioning: a follow-up study

**DOI:** 10.1186/s13054-015-0937-2

**Published:** 2015-04-27

**Authors:** Luuk Wieske, Daniela S Dettling-Ihnenfeldt, Camiel Verhamme, Frans Nollet, Ivo N van Schaik, Marcus J Schultz, Janneke Horn, Marike van der Schaaf

**Affiliations:** Department of Intensive Care Medicine, Academic Medical Center, Meibergdreef 9, 1105 AZ, Amsterdam, The Netherlands; Department of Neurology, Academic Medical Center, Meibergdreef 9, 1105 AZ, Amsterdam, The Netherlands; Department of Rehabilitation, Academic Medical Center, Meibergdreef 9, 1105 AZ, Amsterdam, The Netherlands

## Abstract

**Introduction:**

ICU-acquired weakness is thought to mediate physical impairments in survivors of critical illness, but few studies have investigated this thoroughly. The purpose was to investigate differences in post-ICU mortality and physical functioning between patients with and without ICU-acquired weakness at 6 months after ICU discharge.

**Method:**

ICU patients, mechanically ventilated ≥2 days, were included in a single-center prospective observational cohort study. ICU-acquired weakness was diagnosed when the average Medical Research Council score was <4 in awake and attentive patients. Post-ICU mortality was recorded until 6 months after ICU discharge; in surviving patients, physical functioning was assessed using the Short-Form Health Survey physical functioning domain. The independent effect of ICU-acquired weakness on post-ICU mortality was analyzed using a multivariable Cox proportional hazards model. The independent effect of ICU-acquired weakness on the physical functioning domain score was analyzed using a multivariable linear regression model.

**Results:**

Of the 156 patients included, 80 had ICU-acquired weakness. Twenty-three patients died in the ICU (20 with ICU-acquired weakness); during 6 months follow-up after ICU discharge another 25 patients died (17 with ICU-acquired weakness). Physical functioning domain scores were available for 96 survivors (39 patients with ICU-acquired weakness). ICU-acquired weakness was independently associated with an increase in post-ICU mortality (hazard ratio 3.6, 95% confidence interval, 1.3 to 9.8; *P* = 0.01) and with a decrease in physical functioning (β: -16.7 points; 95% confidence interval, -30.2 to -3.1; *P* = 0.02).

**Conclusion:**

ICU-acquired weakness is independently associated with higher post-ICU mortality and with clinically relevant lower physical functioning in survivors at 6 months after ICU discharge.

**Electronic supplementary material:**

The online version of this article (doi:10.1186/s13054-015-0937-2) contains supplementary material, which is available to authorized users.

## Introduction

After surviving critical illness, many patients suffer from its long-term consequences, which may consist of physical impairments, cognitive dysfunction, and mental health problems [1]. The relevance of physical impairments was described in acute respiratory distress syndrome survivors, who continued to suffer from physical impairments up to 5 years after resolution of critical illness [2]. It is thought that development of ICU-acquired weakness (ICU-AW) is an important mediator of physical impairments [1]. However, evidence supporting this hypothesis is limited [3].

ICU-AW is a frequently occurring neuromuscular complication of critical illness, with an estimated incidence of 46% (95% confidence interval (CI), 43 to 49) in patients with sepsis, prolonged mechanical ventilation or multiple organ dysfunction syndrome [4]. ICU-AW is defined as clinical signs of weakness that develop after the onset of critical illness [5,6]. For diagnosing ICU-AW, muscle strength is assessed manually [5,6]. This can be done reliably when patients are awake and attentive [7]. Weakness may be caused by muscle and/or peripheral nerve dysfunction and damage [5]. If differentiation between these underlying disorders is needed, electrophysiological testing may be performed [5].

In a cohort of acute lung injury survivors, it was found that development of ICU-AW was associated with more physical impairments during follow-up [8]. However, this association was not corrected for possible confounders. Also, acute lung injury survivors represent a subgroup of critically ill patients and the association between ICU-AW and long-term physical impairments in critically ill patients in general remains to be investigated.

Besides being a possible mediator of post-ICU physical impairments, ICU-AW also has a substantial impact on post-ICU mortality. ICU-AW is independently associated with increased in-hospital mortality [9,10]. Also, 1-year mortality is worsened by ICU-AW [11].

In this 6-month follow-up study, we investigated the impact of ICU-AW on the post-ICU period by comparing post-ICU mortality and physical functioning between patients with and without ICU-AW. We hypothesized that ICU-AW is independently associated with increased post-ICU mortality and that survivors with ICU-AW have decreased physical functioning at 6 months after ICU discharge.

## Methods

### Study design and ethical approval

We conducted a single-center prospective observational cohort study. The institutional review board of the Academic Medical Center, Amsterdam, the Netherlands, decided that the study did not fulfill the criteria for medical research as stated in the Dutch ‘Law on medical research’ because the nature of the data collected for this study was judged to be non-intrusive for patients (METC 10/219) and therefore formal informed consent procedures did not apply. Still, we sought verbal and/or written approval of all surviving patients for participating in this study and using their data.

### Study setting

The study was performed in the closed-format tertiary, 34-bed, mixed medical-surgical intensive care unit of the Academic Medical Center in Amsterdam, the Netherlands. As an integral part of care, all patients received early rehabilitation that was continued after transfer to the regular ward until hospital discharge.

### Study population

Inclusion criteria were newly admitted ICU patients aged ≥18 years, mechanically ventilated for ≥2 days. Exclusion criteria were neuromuscular disorders (for example, Guillain-Barré syndrome), any type of stroke, and out-of-hospital cardiac arrest as reasons for admission, and quadriplegia due to a spinal cord syndrome in the medical history or as reason for admission. Additionally, we excluded patients in whom manual muscle strength could not be assessed because of prolonged delirium or failure to awake (that is, up to ICU discharge), patients who had poor functional status before admission (modified Rankin score ≥4 [12]) and patients with a language barrier. Patients with pre-existing neuromuscular disorders not leading to ICU admission or a poor functional status were included in this study. Also, patients admitted because of central nervous system disorders not involving any type of stroke and being awake and alert during ICU admission (for criteria, see below) were included in this study.

### Assessment of ICU-acquired weakness

ICU-AW was diagnosed using the current diagnostic reference standard [5]. As a part of routine care, physical therapists performed manual muscle strength assessments using the Medical Research Council (MRC) score as soon as patients were awake (Richmond Agitation Sedation Scale [13] between -1 and +1) and attentive (able to follow verbal commands using arms or eyelids). MRC scores of six different muscle groups were measured bilaterally; that is, wrist dorsiflexors, elbow flexors, shoulder abductors, hip flexors, knee extensors and ankle dorsiflexors. The scores of muscle groups were summated and divided by the number of muscle groups that could be assessed to obtain an average MRC score (range 0 to 5). Symmetric weakness that had developed after ICU admission with an average MRC score <4 was defined as ICU-AW [5].

### Mortality

All-cause mortality was registered during ICU admission and in the 6 months follow-up after the final ICU discharge date. Mortality of patients who were lost to follow-up was obtained by checking municipal registries.

### Physical functioning

Physical functioning was assessed in patients surviving to 6 months after the final ICU discharge date using the 36-item Short-Form health survey (SF-36) physical functioning (PF) domain score [14,15]. To optimize response rate, the 36-item Short-Form health survey was assessed both by telephone interview conducted by one of the investigators and by mail.

### Baseline and clinical characteristics

During admission, we scored the presence of the following disorders: sepsis [16], severe sepsis [17], septic shock [17] and acute respiratory distress syndrome [18]. Additionally, we collected the following characteristics from the electronic patient file: age, gender, body mass index (kg/m^2^), Charlson co-morbidity index (ranging from 0 to 24 [19]), admission type, Acute Physiology and Chronic Health Evaluation (APACHE) IV score, maximal Sequential Organ Failure Assessment score during admission, days with mechanical ventilation, use of renal replacement therapy, and ICU and hospital length of stay.

### Statistical analysis

The primary analyses were the independent effect of ICU-AW on post-ICU mortality and on the PF domain score. The independent effect of ICU-AW on post-ICU mortality was analyzed using a Cox proportional hazard model (hazard ratio reported with 95% CI) adjusted for confounders. Confounders were *a priori* defined as age, gender, Charlson co-morbidity index, presence of septic shock, APACHE IV score and maximal Sequential Organ Failure Assessment score during admission [20,21]. The independent effect of ICU-AW on the PF domain score was assessed using a multivariable linear regression model adjusted for the above-mentioned confounders (regression coefficient (β) reported with 95% CI). A difference of 10 points on the PF domain score was defined as clinically relevant [22,23]. More detail on statistical methodology and the results of the regression models including the individual variables is provided in Additional file 1. The PF domain score assessed by telephone was used for analysis or, if unavailable, the PF domain score assessed by mail. Agreement between the two interview methods was assessed using the intra-class correlation coefficient (reported with 95% CI) in patients for whom both the telephone and mail PF domain scores were available.

We also analyzed differences in the number of days free from hospital and alive at 3 months after the final ICU discharge between patients with and without ICU-AW using the Wilcoxon rank-sum test.

For descriptive analyses, mean values are presented with standard deviation, median values with interquartile range, and proportions with percentages and total numbers. Differences between proportions were assessed using chi-square test or Fisher’s exact test. Differences between normally distributed variables were assessed using Welch’s *t*-test; differences between non-normally distributed continuous variables were assessed using Wilcoxon rank-sum test. For correlation analyses, spearman’s rho was used in case of ordinal variables and Pearson biserial correlation in case of dichotomous variables.

A *P* value less than 0.05 was considered statistically significant. Analyses were done using R (version: 3.0.1; R Foundation for Statistical Computing, Vienna, Austria).

### Sample size estimation

This study was powered to detect a difference of 10 points on the PF domain score. With an alpha of 0.05, power of 80% and a common standard deviation of 10 [24], 40 patients per group would be needed. Assuming that 50% of newly admitted ICU patients would develop ICU-AW [4], a mortality rate of 35% after 6 months follow-up [25], and that 10% of the population would be lost to follow-up, a population of 140 patients was needed.

## Results

**Figure 1:**
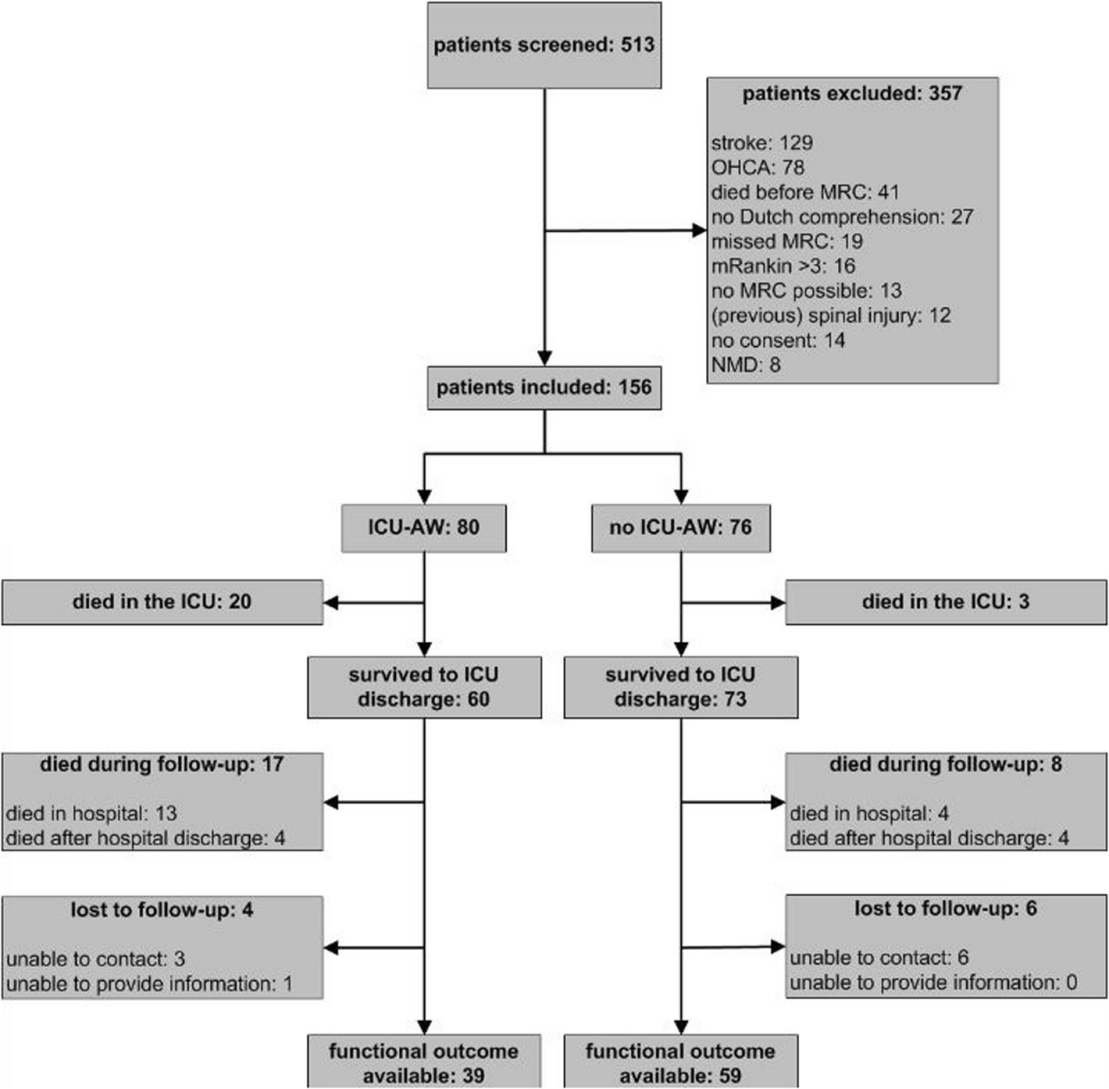
Flowchart of the study population. ICU-AW, ICU-acquired weakness; OHCA, out-of-hospital cardiac arrest; MRC, muscle strength as assessed with Medical Research Council scale; mRankin, modified Rankin score; NMD, neuromuscular disorder.

Between May 2011 and January 2013 156 patients were included, 133 of whom survived to ICU discharge. Figure 1 displays the study flowchart. Patient and admission characteristics are displayed in Table 1.

**Table 1:** **Baseline and ICU admission characteristics for patients surviving to ICU discharge**

	**ICU-AW**	**No ICU-AW**	***P*** **value**
	**(N = 60)**	**(N = 73)**	
**Patient characteristics**			
Age, mean ± SD	65 ± 16	59 ± 14	0.03
Female, n (%)	27 (45)	30 (41)	0.78
BMI (kg/m^2^), mean ± SD	26.8 ± 5.1	26.9 ± 5.2	0.86
BMI >30, n (%)	14 (23)	16 (22)	1.00
Pre-existing neuromuscular disorder, n (%)	3 (5)	1 (1)	0.33
Charlson co-morbidity index, median (IQR)	0 (0-1)	0 (0-2)	0.05
**ICU admission characteristics**			
Admission type			
Medical, n (%)	34 (63)	41 (57)	0.99
Surgical elective, n (%)	15 (21)	18 (24)	
Surgical emergency, n (%)	11 (16)	14 (19)	
APACHE IV score, mean ± SD (2 missing)	81 ± 23	73 ± 28	0.10
CNS disorder as reason of admission, n (%)	1 (2)	0 (0)	0.45
Maximal SOFA score during admission, mean ± SD	12 ± 3	9 ± 4	<0.01
Sepsis during admission, n (%)	56 (93)	57 (78)	0.03
Severe sepsis during admission, n (%)	49 (82)	42 (58)	0.01
Septic shock during admission, n (%)	35 (58)	26 (36)	0.01
Renal replacement therapy during admission, n (%)	23 (38)	19 (26)	0.18
ARDS during admission, n (%)	28 (47)	32 (44)	0.88
Days with mechanical ventilation, median (IQR)	11 (6-17)	5 (4-7)	<0.01
Length of stay in ICU (days), median (IQR)	14 (9-20)	7 (5-10)	<0.01
Average MRC score, median (IQR)	2.8 (1.8-3.5)	4.7 (4-5)	n.a.
**Post-ICU admission characteristics**			
Days free from hospital and alive at 3 months after ICU discharge, median (IQR)	57 (15-71)	75 (56-82)	<0.01
Discharge destination from index hospital if discharged alive			
Other hospital, n/total n (%)	22/53 (41)	14/71 (20)	0.01^*^
Rehabilitation facility, n/total n (%)	14/53 (27)	4/71 (6)	
Home, n/total n (%)	17/53 (32)	53/71 (76)	

APACHE IV, Acute Physiology and Chronic Health Evaluation IV; ARDS, Acute Respiratory Distress Syndrome; BMI, body mass index; CNS, central nervous system; ICU-AW, ICU-acquired weakness; IQR, interquartile range; MRC, muscle strength as assessed with Medical Research Council scale; SD, standard deviation; SOFA, Sequential Organ Failure Assessment score; n.a., not applicable; *, overall test for differences between groups.

### Post-ICU mortality

**Figure 2:**
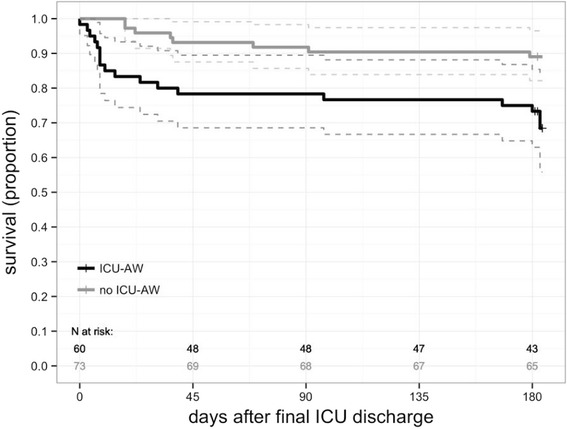
Post-ICU survival curves for patients with and without ICU-acquired weakness. Survival curves for patients with (black line) and without (grey line) intensive care unit-acquired weakness (ICU-AW) starting at final ICU discharge until end of follow-up; that is, 6 months after final ICU discharge. Dotted lines represent the 95% confidence interval; censored patients presented with +.

Post-ICU mortality was higher in patients with ICU-AW (17/60 (28%) vs 8/73 (11%); *P* = 0.02). Lower average MRC scores were correlated with higher mortality (r = -0.21; *P* < 0.01). When adjusted for confounders, ICU-AW was associated with higher post-ICU mortality until 6 months after ICU discharge (hazard ratio 3.6; 95% CI, 1.3 to 9.8; *P* = 0.01; two patients excluded because of missing APACHE IV scores). Figure 2 displays the post-ICU mortality curve. Most deaths occurred during hospital admission; mortality rates after hospital discharge were not different for patients with or without ICU-AW (Table 2). The number of days free from hospital and alive at 3 months after ICU discharge was lower for patients with ICU-AW (Table 1). Overall mortality, including the period of ICU admission, was 37/80 (46%) for patients with ICU-AW and 11/76 (15%) for patients without ICU-AW (*P* < 0.01).

**Table 2:** **Post-ICU outcomes for patients with or without ICU-acquired weakness**

	**ICU-AW**	**No ICU-AW**	***P*** **-value**
Post-ICU mortality			
In-hospital, n/total n (%)	13/60 (22)	4/73 (5)	0.01
After hospital discharge, n/total n (%)	4/47 (9)	4/69 (6)	0.71
PF domain scores at 6 months follow-up, median (IQR; n)	45 (30-70; 39)	75 (50-90; 59)	<0.01
Patients’ residence at 6 months follow-up			
Hospital, n/total n (%)	0/39 (0)	4/59 (7)	0.01*
Rehabilitation facility, n/total n (%)	4/39 (10)	0/59 (0)	
Home, n/total n (%)	35/39 (90)	55/59 (93)	

*Overall *P* value for comparison of all categories. ICU-AW, intensive care unit-acquired weakness; IQR, interquartile range; PF, Short-Form Health Survey physical functioning domain score.

### Physical functioning

Of the 108 patients who survived up to 6 months after ICU discharge, physical functioning was assessed in 98 patients (10 were lost to follow-up; Figure 1). Patients’ place of residence when physical functioning was assessed is shown in Table 2. Sixty-six questionnaires completed via telephone and 32 by mail were used for the analyses. The intra-class correlation coefficient between the telephone-obtained and mail-obtained PF scores was 0.88 (95% CI, 0.67 to 0.95; N = 30). The physical functioning domain score was significantly lower in patients with ICU-AW (Table 2). Higher average MRC scores were correlated with higher physical functioning domain scores (rho = 0.34; *P* < 0.01). After adjusting for confounders, ICU-AW was associated with a decrease of 16.7 points on the PF domain score (95% CI, -30.2 to -3.1; P = 0.02; 2 patients excluded because of missing APACHE IV scores).

## Discussion

The results from this study show that, when assessed at 6 months after ICU discharge, ICU-AW is independently associated with higher post-ICU mortality and with clinically relevant lower physical functioning in survivors.

We found no difference in mortality after hospital discharge. However, it should be noted that, up to the point of hospital discharge, 41% of patients with ICU-AW had already died and the duration of hospital admission after ICU-discharge was much longer for patients with ICU-AW. Hermans and colleagues reported increased 1-year mortality in ICU-AW patients [11]. Additionally, mortality was increased during ICU stay and hospital stay in patients with ICU-AW, which has been reported before [9,10]. Ali and colleagues [9] reported a combined ICU- and in-hospital mortality of 31% in patients with ICU-AW, compared to 6% for patients without ICU-AW. Sharshar and colleagues [10] reported similar mortality rates (31% in patients with ICU-AW; 10% in patients without ICU-AW). Compared to these studies, we found a higher combined ICU- and in-hospital mortality rate in ICU-AW patients (41%), whereas mortality in patients without ICU-AW was similar (9%). Both studies also based the diagnosis of ICU-AW on manual muscle strength with a cut-off of an average MRC score <4. The difference in mortality may be explained by differences in case-mix and inclusion and exclusion criteria.

Increased mortality in ICU-AW may be explained by an increased risk of developing (nosocomial) infections, as was reported by Sharshar and colleagues [10]. The increased risk for developing (nosocomial) infections may be the result of increased durations of mechanical ventilation [26,27], ICU admission [27] and hospital admission [9], which we also found in our study. Alternatively, an increased risk of developing infections may be the result of immune dysfunction secondary to autonomic dysfunction that may accompany ICU-AW [28,29]. Autonomic dysfunction in general may also increase mortality, as was found in critically ill patients [30]. More research is needed to establish reasons for and prevention of mortality in ICU-AW.

In surviving patients, we found that ICU-AW is independently associated with decreased physical functioning. While it was known that survivors with ICU-AW have physical impairments [8,31-33], our study now suggests that, by comparison with critically ill patients without ICU-AW and by correcting for possible confounders, there is an independent effect of ICU-AW on the outcome of critical illness survivors. Moreover, this effect of ICU-AW seems clinically relevant, shown by the difference of more than 10 points on the PF domain score [22,23].

ICU-AW may cause post-ICU physical impairments by various mechanisms. In ICU-AW, muscle and/or nerves can be affected both on a functional and structural level [5]. Decreased excitability may cause muscle and nerve dysfunction and this may resolve quickly [34]. In contrast, structural damage may result in long-term symptoms [34]. Also, nerve involvement as compared to muscle involvement has been linked to worse outcome [32,35,36].

Our study has some limitations. Firstly, because of the single-center design, our results may not be fully generalizable to other populations. The critically ill patients included in this study may represent a relatively healthy population, as shown by the low scores on the Charlson co-morbidity index. Secondly, we did not use electrophysiological studies and muscle biopsies to differentiate between the underlying disorders causing ICU-AW. Three disorders can cause ICU-AW; that is, critical illness polyneuropathy, critical illness myopathy, and critical illness neuromyopathy [5]. The additional diagnostic information obtained by electrophysiology and muscle biopsy is unclear because this has not been studied extensively. Three small studies described a better outcome in critical illness myopathy compared to critical illness polyneuropathy or critical illness neuromyopathy [32,35,36]. Third, our finding that mortality after hospital discharge did not differ between groups may be the result of lack of power because our study was not powered for this analysis. Fourth, due to the nature of the study design, there is the possibility of residual bias confounding the observed association between ICU-AW and physical impairments. Fifth, we did not correct for confounders that function as an intermediate between ICU-AW and physical impairments, such as length of stay in the ICU. Because of ICU-AW, length of stay in the ICU will be longer [35] and the longer length of stay may in turn result in more physical impairments. Instead, we *a priori* selected confounders from the literature that may result in physical impairments in a mechanism independent of ICU-AW. Finally, we did not investigate the impact of the observed physical impairments on (health-related) quality of life of our patients. Quality of life relies on many factors other than physical functioning, such as critical illness-induced cognitive dysfunction or post-traumatic stress disorders, and general factors like age, pre-existing co-morbidities and the availability of support resources and/or caregivers [34,37].

The results of this study have several implications. With a better understanding of the long-term impact of ICU-AW, the prognosis may be discussed more reliably with patients and families by neurologists and intensivists. More information on possibly preventable causes of death in patients with ICU-AW is needed. Furthermore, pathophysiological mechanisms leading to ICU-AW and its long-term sequelae should be clarified to enable interventions preventing or attenuating ICU-AW. Strict glycemic control and late initiation of parenteral nutrition may prevent development of ICU-AW [38,39]. Interventions attenuating the course of ICU-AW are not yet available. Early rehabilitation may be interesting as this has been shown to improve functional outcome in general ICU patients [40].

## Conclusions

We found, in patients mechanically ventilated for 2 days or more, that development of ICU-AW was independently associated with increased post-ICU mortality and clinically relevant lower physical functioning at 6 months after discharge from the ICU. These findings implicate ICU-AW as an important mediator of physical impairments in survivors of critical illness. As such, studies on prevention or treatment of ICU-AW are urgently needed.

## Key messages

- ICU-AW is a frequent complication of critical illness and has been implicated as a mediator of physical impairments in survivors of critical illness. The exact contribution is, however, unknown.
- In this study, we found that ICU-AW was independently associated with clinically relevant lower physical functioning at 6 months after discharge from the ICU.

## Additional file

Additional file 1:
**Statistical analyses and regression models.** Additional methodology describing the statistical analyses as well as the full regression formulae and results.

## Abbreviations

APACHE: Acute Physiology and Chronic Health Evaluation
CI: confidence interval
ICU-AW: ICU-acquired weakness
MRC: Medical Research Council
PF: physical functioning
