# Supplementary material for: Impact of ICU-acquired weakness on post-ICU physical functioning: a follow-up study
Source: Crit Care. 2015 Apr 27;19(1):196. doi: 10.1186/s13054-015-0937-2 (PMC4427976; doi:10.1186/s13054-015-0937-2)
Supplement: Additional file 1: — Statistical analyses and regression models. Additional methodology describing the statistical analyses as well as the full regression formulae and results. [file 13054_2015_937_MOESM1_ESM.pdf]

# Impact of ICU-acquired weakness on post-ICU physical functioning: a follow-up study

Luuk Wieske, Daniela S. Dettling-Ihnenfeldt, Camiel Verhamme, Frans Nollet, Ivo N. van Schaik, Marcus J. Schultz, Janneke Horn and Marike van der Schaaf

## Additional file 1

Contents:

1. Statistical analyses

2. Regression models

## 1. Statistical analyses

*Propensity score*

Because of the sample size, all a priori defined confounders for the multivariate models were summarized into a propensity score. The propensity score was calculated using a logistic regression model with ICU-AW as the dependent variable and the confounders as the independent variables. The resulting predicted value per patient was transformed to the probability scale making the propensity score. The propensity was subsequently added as an independent variable along with ICU-AW in the multivariable analyses.

*Multivariable models*

Two different multivariable models were used in this study: i.e. a multivariable Cox proportional hazard model to investigate the independent effect of ICU-AW on post-ICU mortality and a multivariable linear regression model to investigate he independent effect of ICU-AW on the physical functioning (PF) domain score.

For the multivariable Cox model, the time to event was first calculated from the day of ICU discharge to day of death up to six months after ICU discharge. Follow-up data for mortality was complete for all patients. Next, the propensity score and ICU-AW were entered in the Cox model to obtain an adjusted hazard ratio. The proportionality of the hazard was verified.

For the multivariable linear regression model, the PF domain score was used as the dependent variable. Although not truly a continuous dependent variable, model assumptions of normality were not violated. ICU-AW and the propensity score were entered as independent variables to the model. The mean predicted effect of

ICU-AW on the PF domain score (the regression coefficient (β) reported with 95% confidence interval) was used for reporting.

## 2. Regression models

*Propensity score*

The following regression formula was used to calculate the propensity score *"propensity score = predict(lrm( ICUAW~ age+ gender+ CCI+ max.sofa+ apacheIV+ sep.shock), type="fitted")"*. The following model was produced:

| Variable | Regression coefficient | p-value |
|---|---|---|
| intercept | -4.736 | <0.01 |
| age | 0.034 | 0.02 |
| gender=female | 0.409 | 0.28 |
| CCI | -0.332 | <0.01 |
| max.sofa | 0.274 | <0.01 |
| apacheIV | -0.004 | 0.61 |
| sep.shock=yes | 0.280 | 0.50 |
| **model likelihood ratio test ($X^2$)** | **41.53 (6 df)** | **<0.01** |

CCI: Charlson co-morbidty index; SOFA: Sequential Organ Failure Score; apacheIV: Acute Physiology and Chronic Health Evaluation IV score; df: degrees of freedom

*Multivariable Cox proportional hazard model*

The following regression formula was used to calculate the hazard ratio *"hazard ratio = coxph(surv.obj (observation time) ~ propensity score*+ *ICUAW)"*. The following model was produced:

| **Variable** | **Regression coefficient** | **p-value** |
|---|---|---|
| propensity score | -0.212 | 0.83 |
| ICUAW | 1.292 | 0.01 |
| **model likelihood ratio test ($X^2$)** | **8.62 (2 df)** | **0.01** |

ICUAW: Intensive Care Unit – acquired weakness; df: degrees of freedom

*Multivariable linear regression model*

The following regression formula was used to calculate the hazard ratio *"regression coefficient = lm(SF36 physical functioning domain score ~ propensity score*+ *ICUAW)"*. The following model was produced:

| **Variable** | **Regression coefficient** | **p-value** |
|---|---|---|
| propensity score | -6.868 | 0.61 |
| ICUAW | -16.684 | 0.02 |
| **F-statistic** | **4.499 (2 df)** | **0.01** |

ICUAW: Intensive Care Unit – acquired weakness; df: degrees of freedom
